# Safety, immunogenicity and persistence of immune response to the combined diphtheria, tetanus, acellular pertussis, poliovirus and *Haemophilus influenzae* type b conjugate vaccine (DTPa-IPV/Hib) administered in Chinese infants

**DOI:** 10.1080/21645515.2016.1239670

**Published:** 2016-10-21

**Authors:** Yanping Li, Rong Cheng Li, Qiang Ye, Changgui Li, You Ping Liu, Xiao Ma, Yanan Li, Hong Zhao, Xiaoling Chen, Deepak Assudani, Naveen Karkada, Htay Htay Han, Olivier Van Der Meeren, Narcisa Mesaros

**Affiliations:** a Guangxi Zhuang Autonomous Region Center for Disease Control and Prevention, Nanning City, Guangxi, China; b National Institutes for Food and Drug Control, Beijing, China; c Wuzhou Center for Disease Control and Prevention, Wuzhou City, Guangxi, China; d Mengshan Center for Disease Control and Prevention, Disease Prevention, Development District, Wuzhou City, Guangxi, China; e GSK, Bangalore, India; f GSK, King of Prussia, PA, USA; g GSK, Wavre, Belgium

**Keywords:** acellular pertussis, conjugate vaccine, diphtheria, DTPa-IPV/Hib, *Haemophilus influenzae* type b, immunogenicity, infants, poliovirus, safety, tetanus

## Abstract

We conducted 3 phase III, randomized, open-label, clinical trials assessing the safety, reactogenicity (all studies), immunogenicity (Primary vaccination study) and persistence of immune responses (Booster study) to the combined diphtheria, tetanus, pertussis, poliomyelitis, and *Haemophilus influenzae* type b vaccine (DTPa-IPV/Hib) in Chinese infants and toddlers.

In the Pilot study (NCT00964028), 50 infants (randomized 1:1) received 3 doses of DTPa-IPV/Hib at 2–3–4 (Group A) or 3–4–5 months of age (Group B). In the Primary study (NCT01086423), 984 healthy infants (randomized 1:1:1) received 3 doses of DTPa-IPV/Hib at 2–3–4 (Group A) or 3–4–5 (Group B) months of age, or concomitant DTPa/Hib and poliomyelitis (IPV) vaccination at 2–3–4 months of age (Control group); 825 infants received a booster dose of DTPa/Hib and IPV at 18–24 months of age (Booster study; NCT01449812).

In the Pilot study, unsolicited symptoms were more frequent in Group A (16 versus 1 infant; mostly upper respiratory tract infection and pyrexia); this observation was attributed to an epidemic outbreak of viral infections. Non-inferiority of 3-dose primary vaccination with DTPa-IPV/Hib over separately administered DTPa/Hib and IPV was demonstrated for Group A (primary objective). Similar antibody concentrations were observed in all groups, except for anti-polyribosyl-ribitol phosphate and anti-poliovirus types 1–3 which were higher in DTPa-IPV/Hib recipients. Protective antibody levels against all vaccine antigens remained high until booster vaccination. Three-dose vaccination with DTPa-IPV/Hib had a clinically acceptable safety profile.

## Introduction

Vaccination has led to a significant reduction in the number of serious childhood diseases, such as diphtheria, tetanus, pertussis, invasive *Haemophilus influenzae* type b (Hib) disease, and poliomyelitis (also commonly called polio), which are associated with significant levels of morbidity and mortality. The routine use of vaccines to protect against these diseases, recommended by the World Health Organization (WHO) is well established globally. However, despite the wide vaccine coverage, the burden of these diseases remains high, particularly in developing countries.^1^

In China, vaccination against diphtheria, pertussis and tetanus is mandatory for all infants under the National Expanded Program on Immunization (EPI) since 1960s. In 2006, vaccination coverage against the 3 diseases reached 99.0%, and since 2006, the total annual incidence of reported cases of diphtheria, pertussis and tetanus decreased to below 0.5 cases per 100,000 population.^2^ The combined diphtheria, tetanus and acellular pertussis vaccine (DTPa) is recommended for administration as 3-dose primary vaccination at 3, 4 and 5 months of age, and a fourth (booster) dose between 18 and 24 months.^3^

Hib is a leading cause of childhood bacterial meningitis, pneumonia, and other serious infections, which can be almost completely eliminated through routine vaccination. In China, the Hib conjugate vaccine has been available since 2000; however, the lack of formal national recommendation for its use affects the vaccination coverage, and an estimated 19,000 childhood deaths from Hib occur each year in China.^4^

The widespread use of oral poliovirus vaccine (OPV) and inactivated poliovirus vaccine (IPV) has led to a drastic reduction in the incidence of polio, which has been eradicated from the Americas, the Western Pacific, and the European WHO regions.^1^ Although OPV has been the mainstay of poliomyelitis control in many countries since the 1950s, it may rarely cause vaccine associated paralytic poliomyelitis, due to reverse mutations in the RNA genome of the attenuated vaccine strains resulting in neurovirulence.^5^ In countries where OPV is used in routine immunization programs, outbreaks of poliomyelitis caused by circulating vaccine-derived strains remain a potential threat. In China, the last case of domestic wild-type poliomyelitis was reported in 1994, and the country was certified polio-free by the WHO in 2000.^6^ However, several outbreaks of vaccine-derived poliovirus infections have been reported during the last decade.^7-9^ The inactivation of the virus in IPV prevents reverse mutations and neurovirulence. The current Chinese poliomyelitis immunization schedule comprises 3 doses of OPV at 2, 3 and 4 months of age, with one booster dose at 4 y of age. In recently conducted clinical trials with IPV (*Poliorix*™, GSK, Belgium) in China, the vaccine was well tolerated and immunogenic when administered in infants according to the Chinese primary vaccination immunization schedule, and as a booster in the second year of life.^10^

The EPI in China covers 12 pediatric diseases, including diphtheria, tetanus, pertussis and polio, with up to 25 administrations of 12 vaccines recommended in a child's first 18–24 months of life.^11^ However, the complex logistics related to the administration of different multi-dose vaccines may affect the compliance and acceptance related to vaccine administration. Combination vaccines allow the administration of antigens that target multiple diseases in a single injection, offering significant advantages over the single vaccines such as increased patient and health care acceptance, higher vaccination coverage, reduction in number of visits and the related costs, and minimized risk of administration errors and missed doses.^12^

A combined pentavalent vaccine against diphtheria, tetanus, pertussis, poliomyelitis, and Hib (DTPa-IPV/Hib; *Infanrix™-IPV/Hib* GSK, Belgium) has been first licensed in 1997.^13^ The vaccine administered as a primary and/or booster vaccination has been shown to be well tolerated and immunogenic in previous studies conducted in infants in other countries outside China.^14-19^

Three clinical trials were undertaken to assess the safety, reactogenicity and immunogenicity of the DTPa-IPV/Hib vaccine in Chinese infants. The first (Pilot) study, evaluated the safety and reactogenicity of DTPa-IPV/Hib given as a single injection to Chinese infants at 2, 3 and 4 months of age or at 3, 4 and 5 months of age. The immunogenicity, safety and reactogenicity of the vaccine were then assessed in a Primary vaccination study, and the persistence of immune response to the vaccine in a follow-up, Booster vaccination study.

## Results

### Study participants

In the Pilot study, 50 infants were included in the total vaccinated cohort (TVC); 25 received the DTPa-IPV/Hib vaccine at 2, 3, 4 months of age (Group A), and 25 received the vaccine at 3, 4, 5 months of age (Group B). Forty-nine infants completed the study; for one infant, the consent was withdrawn, but not due to an adverse event (AE) or a serious AE (SAE). The mean age of infants was 12.1 (±2.40) weeks; 64.0% were male (Table 1). All infants were of Asian - East Asian heritage.

**Table 1.** Summary of demographic characteristics (Total vaccinated cohorts).

***Pilot study*Characteristics**	**Group A(N = 25)**	**Group B(N = 25)**	**Total (N = 50)**	
Age (weeks), mean (SD)	10.1 (1.36)	14.2 (1.18)	12.1 (2.40)	
Gender				
Female, n (%)	7 (28.0)	11 (44.0)	18 (36.0)	
Male, n (%)	18 (72.0)	14 (56.0)	32 (64.0)	
***Primary vaccination study***				
**Characteristics**	**Group A(N = 330)**	**Group B(N = 324)**	**Control(N = 330)**	**Total(N = 984)**
Age (weeks), mean (SD)	9.9 (1.12)	14.3 (1.14)	9.9 (1.17)	11.3 (2.37)
Gender				
Female, n (%)	155 (47.0)	147 (45.4)	141 (42.7)	443 (45.0)
Male, n (%)	175 (53.0)	177 (54.6)	189 (57.3)	541 (55.0)
Booster vaccination study^*^				
**Characteristics**	**Group A(N = 272)**	**Group B(N = 273)**	**Control(N = 280)**	**Total(N = 825)**
Age (weeks), mean (SD)	19.5 (0.9)	19.4 (0.9)	19.5 (1.0)	19.5 (0.9)
Gender				
Female, n (%)	131 (48.2)	126 (46.2)	120 (42.9)	377 (45.7)
Male, n (%)	141 (51.8)	147 (53.8)	160 (57.1)	448 (54.3)

N, total number of participants; n (%), number (percentage) of participants in a given category; SD, standard deviation.

Group A, infants who received DTPa-IPV/Hib vaccine at 2, 3, 4 months of age.

Group B, infants who received DTPa-IPV/Hib vaccine at 3, 4, 5 months of age.

Control, infants who received DTPa/Hib and IPV vaccines at 2, 3, 4 months of age.

* In the Booster study, all children received booster vaccination with the same vaccines, i.e. DTPa/Hib and IPV.

**Figure 1.**
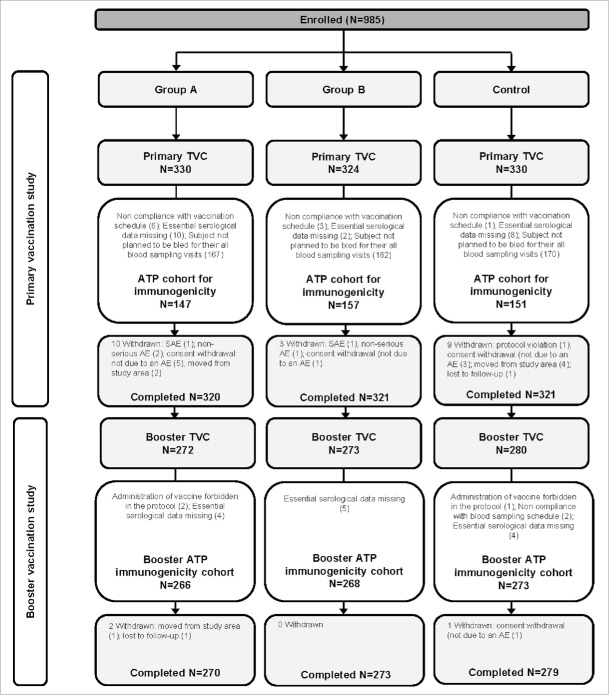
Participant flow in the Primary and Booster vaccination studies. Group A, infants who received DTPa-IPV/Hib vaccine at 2, 3, 4 months of age; Group B, infants who received DTPa-IPV/Hib vaccine at 3, 4, 5 months of age; Control, infants who received DTPa/Hib and IPV vaccines at 2, 3, 4 months of age N, number of participants; TVC, total vaccinated cohort; ATP, according-to-protocol; SAE, serious adverse event; AE, adverse event. One participant was not included in the TVC due to consent withdrawal before vaccination.

A total of 985 infants were enrolled in the Primary study; 984 were included in the TVC and 962 completed the study (Fig. 1). The according-to-protocol (ATP) cohort for immunogenicity included 455 infants: 147 received DTPa-IPV/Hib vaccine at 2, 3, 4 months of age (Group A), 157 received the vaccine at 3, 4, 5 months of age (Group B), and 151 received the concomitant DTPa/Hib (*Infanrix*™ *Hib*; GSK, Belgium) and IPV (*Poliorix*™, GSK, Belgium) at 2, 3, 4 months of age (Control group). The mean age was 11.3 (±2.37) weeks and 55.0% were male (Table 1).

A total of 825 infants were included in the TVC of the Booster study (272 in Group A, 273 in Group B, and 280 in the Control group), in which all infants received booster dose with the same vaccines, DTPa/Hib and IPV; 822 infants completed the study, and 807 were included in the ATP cohort for immunogenicity (Fig. 1). The mean age of infants was 19.5 (±0.9) months and 54.3% were male (Table 1). The ATP cohort for immunogenicity in the Primary study included a lower number of participants compared to the Booster study, as in the Primary study immunogenicity was assessed only in a subset of participants, while in the Booster study, all eligible participants were included.

All infants included in the Primary and Booster studies were of Asian - Chinese heritage.

### Safety

**Figure 2.**
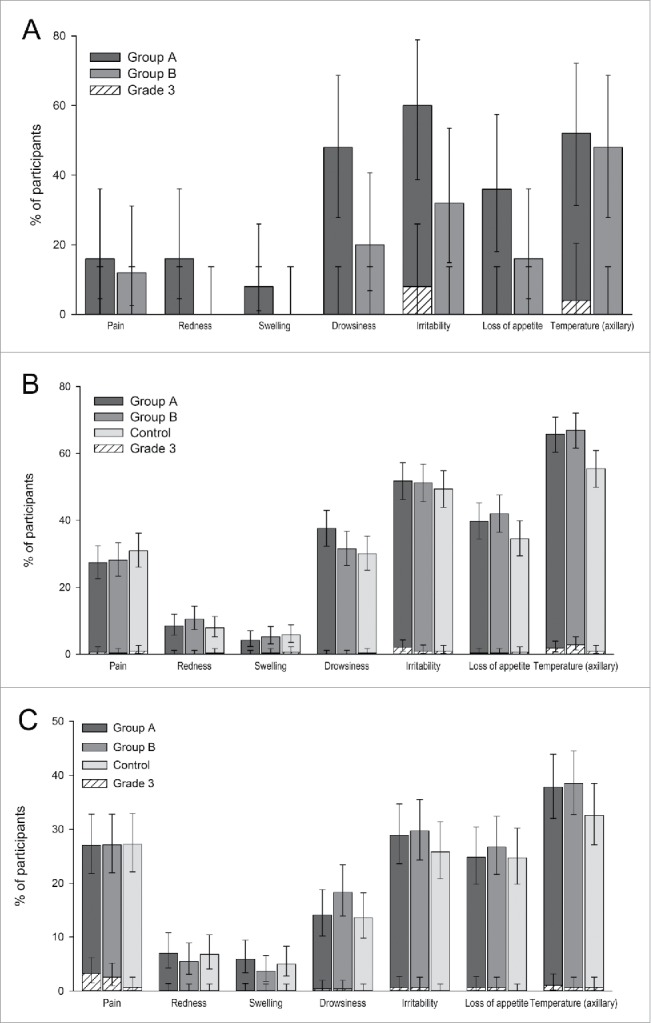
Solicited local and general symptoms reported within 4 d (day 0–3) after vaccination with DTPa-IPV/Hib in the Pilot study (A), or with DTPa-IPV/Hib or DTPa/Hib and IPV in the Primary vaccination study (B) or with or DTPa/Hib and IPV in the Booster vaccination study (C) (Total vaccinated cohorts). Group A, infants who received DTPa-IPV/Hib vaccine at 2, 3, 4 months of age; Group B, infants who received DTPa-IPV/Hib vaccine at 3, 4, 5 months of age; Control, infants who received DTPa/Hib and IPV vaccines at 2, 3, 4 months of age; %, percentage of participants. The error bars indicate 95% confidence intervals.

In the Pilot study, solicited local and general symptoms were reported for 24.0% and 80.0% of infants in group A and for 12.0% and 60.0% of infants in Group B. The most frequently reported solicited local symptom was pain at the injection site, reported in 16.0% of infants from Group A and 12.0% of infants from Group B; no grade 3 solicited local symptoms were reported. The most frequently reported solicited general symptoms were irritability, reported in 60.0% of infants from Group A, and fever, reported in 48.0% of infants from Group B. The most common grade 3 solicited general symptom was irritability reported in 8.0% of infants from Group A (Fig. 2). Irritability and fever were also the most frequently reported solicited general symptoms assessed by the investigator to be causally related to vaccination, with irritability reported in 52.0% and 32.0% of infants, and fever reported in 48.0% and 44.0% of infants in Groups A and B, respectively. At least one unsolicited symptom was reported in 64.0% of infants in Group A and 4.0% of infants in Group B; the most frequently reported was upper respiratory tract infection, reported in 40.0% of infants from Group A and 4.0% of infants from Group B. Only one grade 3 unsolicited symptom was reported in Group A (pyrexia). Unsolicited symptoms considered by the investigator as causally related to vaccination were reported in 12.0% of infants from Group A; no unsolicited symptoms with a possible causal relationship to vaccination were reported in Group B. No SAEs or deaths were reported.

In the Primary vaccination study, the most frequently reported solicited local symptom was pain at the injection site, reported in 27.3% of infants from Group A, 28.1% of infants from Group B, and 30.9% of infants from the Control group; grade 3 pain was reported in 0.6%, 0.3%, and 0.9% of infants in these groups, respectively (Fig. 2). Fever was the most frequently reported solicited general symptom in all the 3 groups (Group A: 65.8%, Group B: 67.0% and Control: 55.5% of infants). The most common grade 3 general symptoms were irritability and fever (irritability: 2.1%, 0.9% and 0.9%, and fever: 1.8%, 2.8% and 0.9% of infants in groups A, B, and Control, respectively; Fig. 2). At least one unsolicited symptom was reported in 29.7%, 35.2%, and 33.3% of infants in Groups A, B and Control, respectively; the most common was nasopharyngitis, reported in 12.7%, 14.2%, and 15.2% of infants in these groups, respectively. Grade 3 unsolicited symptoms were reported for 1.5% of infants in Group A, 0.9% of infants in Group B and 0.3% of infants in the Control group; none of the grade 3 unsolicited symptoms were assessed by the investigator to be causally related to vaccination. Unsolicited symptoms that were considered by the investigator to be causally related to vaccination were reported for 2.7% of infants in Group A, 1.2% of infants in Group B and 3.0% of infants in the Control group. A total of 17 SAEs were reported in 13 infants, 6 in Group A, 3 in Group B and 4 in the Control group. One SAE (diarrhea; Control group) was considered by the investigator as causally related to the vaccination. All except 2 SAEs (hypokalaemia and malnutrition) resolved by the end of the study. One infant died during the course of the study; this 4-month-old infant from Group A developed acute bronchopneumonia, hypokalemia and protein malnutrition one day after the third dose of the DTPa-IPV/Hib vaccine. The cause of death was infectious shock due to acute bronchopneumonia and congestive heart failure. These two events were not considered by the investigator as causally related to the vaccination.

In the Booster study, pain at the injection site was the most common solicited local symptom, reported in approximately 27.0% of infants in each group, and the most common grade 3 symptom, reported in 3.3%, 2.6%, and 0.7% of infants from groups A, B, and Control, respectively. The most common general symptom in all groups was fever, reported in 37.8%, 38.5%, and 32.6% of infants in these groups, respectively (Fig. 2). At least one unsolicited symptom was reported in 5.9%, 4.8%, and 7.5% of infants from groups A, B, and Control, respectively; the most common unsolicited symptom was nasopharyngitis, reported for 2.9%, 2.2%, and 4.6% of infants in these groups, respectively. Three grade 3 unsolicited symptoms (bronchitis, pharyngitis and upper respiratory tract infection) were reported in 3 infants from the Control group. One unsolicited symptom, abdominal pain (Group A) was considered by the investigator to be causally related to vaccination.

Two SAEs, bronchopneumonia and febrile convulsions, were reported in one infant; both resolved and were not considered as related to vaccination. No fatal SAEs were reported.

### Immunogenicity

#### Primary vaccination study

The primary objective of non-inferiority for the DTPa-IPV/Hib vaccine compared to separately administered DTPa/Hib and IPV vaccines was met, as the upper limits (ULs) of the standardized asymptotic 95% confidence intervals (CIs) for the difference between groups (Control group minus Group A) in terms of seroprotection rates for anti-diphtheria toxoid (DT), anti-tetanus toxoid (TT), anti-polyribosyl-ribitol phosphate (PRP) and anti-poliovirus types 1, 2 and 3 antibodies, and in terms of percentage of participants with a vaccine response to pertussis antigens, were below the pre-defined limit of 10% for all antigens: anti-DT (difference: 0.68%, UL: 3.76), anti-TT (0.00%, 2.56), anti-PRP (−7.93%, −2.13), anti-poliovirus types 1, 2 and 3 (0.00%, 2.56 for each antigen), anti-pertussis toxoid (PT; −0.68%, 1.89), anti-filamentous hemagglutinin (FHA; −2.70%, −0.11) and anti-pertactin (PRN; −0.67%, 3.04). Vaccine response was defined as anti-PT and anti-FHA antibody concentration ≥20 enzyme-linked immunosorbent assay (ELISA) units (EU)/ml one month after the third vaccine dose, anti-PRN antibody concentration ≥20 EU/ml (4-fold the assay cut-off) one month after the third vaccine dose for initially seronegative participants, and at least a 4-fold increase in antibody concentration from pre to post-vaccination for initially seropositive participants.

One month after the primary vaccination, all infants in the 3 groups had seroprotective concentrations of anti-TT (≥0.1 international units [IU]/ml) and anti-poliovirus types 1, 2, and 3 (≥ 8 median effective dose [ED_50_]) antibodies, at least 99.3% of infants had seroprotective concentrations of anti-DT (≥0.1 IU/ml) antibodies, at least 88.7% had seroprotective concentrations of anti-PRP antibodies (≥0.15 µg/ml), and all infants in Groups A and B and all except one in the Control group (99.3%) were seropositive for anti-PT, anti-FHA and anti-PRN antibodies (≥5 EU/ml) (Table 2). One month after the third dose of the primary vaccination, anti-DT and anti-TT antibody geometric mean concentrations (GMCs) were similar across the 3 groups and ranged from 0.613–0.753 EU/ml for anti-DT, and 3.618–4.124 EU/ml for anti-TT. Anti-PRP GMCs seemed higher in DTPa-IPV/Hib recipients (Groups A and B; 5.601–9.396 EU/ml) compared to control (2.826 EU/ml). Similar trend was observed for anti-poliovirus type 1, 2, and 3 antibodies, with geometric mean titers (GMTs) in DTPa-IPV/Hib recipients ranging from 1143.7–1328.9 ED_50_, 416.2–458.6 ED_50_, and 1411.6–1478.8 ED_50_ for these 3 types, compared to GMTs of 533.6 ED_50_, 186.4 ED_50_, and 820.7 ED_50_, respectively, in the control group. The response to pertussis antigens was comparable between the groups, with GMCs ranging from 97.1–114.7 EU/ml, 76.3–87.7 EU/ml, and 43.2–44.8 EU/ml for anti-PT, anti-FHA, and anti-PRN antibodies, respectively (Table 2).

**Table 2.** Seroprotection/seropositivity rates and geometric mean antibody concentrations/titers before and one month after third dose/booster with DTPa-IPV/Hib or DTPa/Hib and IPV vaccines in the Primary and Booster vaccination studies (ATP cohort for immunogenicity).

			Primary Vaccination						Booster Vaccination^*^			
Antibody	Groups	Timepoint	N	n (%)	95% CI	GMC/GMT	95% CI	N	n (%)	95% CI	GMC/GMT	95% CI
Anti-DT(≥0.1 IU/ml)	Group A	PRE	147	3 (2.0)	0.4–5.8	0.052	0.050–0.053	265	235 (88.7)	84.2–92.2	0.174	0.162–0.187
		POST	147	146 (99.3)	96.3–100.0	0.719	0.661–0.782	265	265 (100.0)	98.6–100.0	1.341	1.239–1.451
	Group B	PRE	157	2 (1.3)	0.2–4.5	0.051	0.050–0.052	267	245 (91.8)	87.8–94.8	0.189	0.176–0.202
		POST	156	156 (100.0)	97.7–100.0	0.753	0.699–0.812	268	268 (100.0)	98.6–100.0	1.504	1.377–1.643
	Control	PRE	151	1 (0.7)	0.0–3.6	0.051	0.049–0.052	272	228 (83.8)	78.9–88.0	0.154	0.142–0.166
		POST	147	147 (100.0)	97.5–100.0	0.613	0.565–0.666	270	270 (100.0)	98.6–100.0	1.227	1.134–1.326
Anti-TT(≥0.1 IU/ml)	Group A	PRE	147	2 (1.4)	0.2–4.8	0.051	0.050–0.052	266	264 (99.2)	97.3–99.9	0.455	0.429–0.483
		POST	147	147 (100.0)	97.5–100.0	4.118	3.779–4.488	266	266 (100.0)	98.6–100.0	4.862	4.614–5.124
	Group B	PRE	157	4 (2.5)	0.7–6.4	0.052	0.050–0.054	268	266 (99.3)	97.3–99.9	0.511	0.482–0.542
		POST	156	156 (100.0)	97.7–100.0	4.124	3.796–4.479	268	268 (100.0)	98.6–100.0	4.927	4.693–5.173
	Control	PRE	151	0 (0.0)	0.0–2.4	0.050	0.050–0.050	271	268 (98.9)	96.8–99.8	0.380	0.357–0.403
		POST	147	147 (100.0)	97.5–100.0	3.618	3.339–3.921	272	272 (100.0)	98.7–100.0	4.371	4.161–4.591
Anti-PRP(≥0.15 µg/ml)	Group A	PRE	146	25 (17.1)	11.4–24.2	0.127	0.104–0.154	266	221 (83.1)	78.0–87.4	2.308	1.878–2.836
		POST	147	142 (96.6)	92.2–98.9	5.601	4.676–6.709	266	264 (99.2)	97.3–99.9	35.178	30.617–40.418
	Group B	PRE	157	32 (20.4)	14.4–27.5	0.135	0.112–0.163	268	229 (85.4)	80.6–89.4	2.743	2.245–3.352
		POST	157	155 (98.7)	95.5–99.8	9.396	8.032–10.992	268	268 (100.0)	98.6–100	49.023	43.649–55.058
	Control	PRE	151	35 (23.2)	16.7–30.7	0.150	0.122–0.185	273	234 (85.7)	81.0–89.6	2.407	1.993–2.908
		POST	150	133 (88.7)	82.5–93.3	2.826	2.235–3.572	273	271 (99.3)	97.4–99.9	27.682	24.251–31.598
Anti-PT(≥5 EU/ml)	Group A	PRE	147	23 (15.6)	10.2–22.5	2.9	2.8–3.1	266	254 (95.5)	92.3–97.6	10.3	9.5–11.1
		POST	147	147 (100.0)	97.5–100.0	108.7	99.2–119.1	266	266 (100.0)	98.6–100.0	138.5	132.0–145.3
	Group B	PRE	157	11 (7.0)	3.5–12.2	2.7	2.6–2.8	268	258 (96.3)	93.2–98.2	12.2	11.3–13.1
		POST	156	156 (100.0)	97.7–100.0	114.7	105.4–124.8	268	268 (100.0)	98.6–100.0	146.2	139.7–153.0
	Control	PRE	151	16 (10.6)	6.2–16.6	2.8	2.6–2.9	273	253 (92.7)	88.9–95.5	10.3	9.5–11.2
		POST	148	147 (99.3)	96.3–100.0	97.1	88.3–106.8	273	273 (100.0)	98.7–100.0	126.8	120.4–133.5
Anti-FHA(≥5 EU/ml)	Group A	PRE	147	19 (12.9)	8.0–19.4	2.9	2.7–3.1	266	256 (96.2)	93.2–98.2	12.7	11.8–13.6
		POST	147	147 (100.0)	97.5–100.0	87.7	79.9–96.3	266	266 (100.0)	98.6–100.0	124.6	119.2–130.2
	Group B	PRE	157	9 (5.7)	2.7–10.6	2.6	2.5–2.7	268	262 (97.8)	95.2–99.2	14.3	13.4–15.2
		POST	156	156 (100.0)	97.7–100.0	87.6	79.6–96.4	268	268 (100.0)	98.6–100.0	124.0	119.2–129.0
	Control	PRE	151	24 (15.9)	10.5–22.7	2.9	2.7–3.1	273	255 (93.4)	89.8–96.0	12.3	11.4–13.3
		POST	148	147 (99.3)	96.3–100.0	76.3	68.5–85.0	273	273 (100.0)	98.7–100.0	120.8	115.3–126.6
Anti-PRN(≥5 EU/ml)	Group A	PRE	147	6 (4.1)	1.5–8.7	2.6	2.5–2.7	266	254 (95.5)	92.3–97.6	9.2	8.7–9.6
		POST	147	147 (100.0)	97.5–100.0	44.8	42.0–47.9	266	266 (100.0)	98.6–100.0	57.3	55.6–59.1
	Group B	PRE	157	4 (2.5)	0.7–6.4	2.6	2.5–2.6	268	260 (97.0)	94.2–98.7	9.7	9.2–10.2
		POST	156	156 (100.0)	97.7–100.0	43.7	41.3–46.3	268	268 (100.0)	98.6–100.0	59.9	58.1–61.8
	Control	PRE	151	3 (2.0)	0.4–5.7	2.5	2.5–2.6	273	260 (95.2)	92.0–97.4	9.0	8.5–9.5
		POST	148	147 (99.3)	96.3–100.0	43.2	39.8–47.0	273	273 (100.0)	98.7–100.0	57.2	55.3–59.1
Anti-poliovirus type 1	Group A	PRE	146	63 (43.2)	35.0–51.6	9.4	7.7–11.5	266	253 (95.1)	91.8–97.4	72.0	62.0–83.7
		POST	147	147 (100.0)	97.5–100.0	1143.7	952.7–1372.9	265	265 (100.0)	98.6–100.0	3512.2	3159.7–3904.1
	Group B	PRE	157	62 (39.5)	31.8–47.6	7.1	6.2–8.2	268	262 (97.8)	95.2–99.2	96.3	82.8–112.0
		POST	157	157 (100.0)	97.7–100.0	1328.9	1137.6–1552.4	268	268 (100.0)	98.6–100.0	3410.9	3081.7–3775.4
	Control	PRE	151	69 (45.7)	37.6–54.0	9.2	7.7–11.0	273	263 (96.3)	93.4–98.2	75.9	65.7–87.6
		POST	150	150 (100.0)	97.6–100.0	533.6	469.5–606.4	273	273 (100.0)	98.7–100.0	3386.8	3078.0–3726.6
Anti-poliovirus type 2	Group A	PRE	146	43 (29.5)	22.2–37.6	6.3	5.5–7.2	266	243 (91.4)	87.3–94.4	56.5	46.2–69.0
		POST	147	147 (100.0)	97.5–100.0	416.2	344.5–502.8	265	265 (100.0)	98.6–100.0	1931.2	1721.7–2166.2
	Group B	PRE	157	32 (20.4)	14.4–27.5	5.0	4.6–5.5	268	256 (95.5)	92.3–97.7	64.1	53.8–76.4
		POST	157	157 (100.0)	97.7–100.0	458.6	385.6–545.5	268	268 (100.0)	98.6–100.0	2237.9	2001.6–2502.1
	Control	PRE	151	54 (35.8)	28.1–44.0	6.9	6.0–8.0	273	244 (89.4)	85.1–92.8	41.9	35.0–50.0
		POST	150	150 (100.0)	97.6–100.0	186.4	160.4–216.5	273	273 (100.0)	98.7–100.0	1886.1	1679.6–2117.9
Anti-poliovirus type 3	Group A	PRE	146	29 (19.9)	13.7–27.3	5.8	5.0–6.8	266	249 (93.6)	90.0–96.2	72.6	61.1–86.3
		POST	147	147 (100.0)	97.5–100.0	1478.8	1210.6–1806.5	265	265 (100.0)	98.6–100.0	5237.8	4671.8–5872.3
	Group B	PRE	157	18 (11.5)	6.9–17.5	4.9	4.3–5.6	268	254 (94.8)	91.4–97.1	79.4	65.8–95.8
		POST	157	157 (100.0)	97.7–100.0	1411.6	1175.3–1695.3	268	268 (100.0)	98.6–100.0	5438.5	4846.8–6102.4
	Control	PRE	151	34 (22.5)	16.1–30.0	5.7	5.1–6.4	273	250 (91.6)	87.6–94.6	60.3	50.8–71.6
		POST	150	150 (100.0)	97.6–100.0	820.7	698.5–964.4	273	273 (100.0)	98.7–100.0	5141.2	4650.1–5684.2

Group A, infants who received the DTPa-IPV/Hib vaccine at 2, 3, 4 months of age in the Primary study; Group B, infants who received DTPa-IPV/Hib vaccine at 3, 4, 5 months of age in the Primary study; Control, infants who received DTPa/Hib and IPV vaccines separately at 2, 3, 4 months of age in the Primary study; n, number of infants with concentration equal to or above the specified value; %, percentage of infants with concentration equal to or above the specified value; ATP, according-to-protocol; PRE, pre-vaccination blood sampling timepoint; POST, post-vaccination blood sampling timepoint, one month after the third dose; 95% CI, 95% confidence interval; LL, lower limit; UL, upper limit; GMT, geometric mean titer; GMC, geometric mean concentration; anti-DT, anti-diphtheria toxoid; anti-TT, anti-tetanus toxoid; anti-PRP, anti-polyribosyl-ribitol-phosphate; anti-PT, anti-pertussis toxoid; anti-FHA, anti-filamentous hemagglutinin; anti-PRN, anti-pertactin; IU/ml, international units per milliliter; EU/ml, ELISA units per milliliter.

* In the Booster study, all children received booster vaccination with the same vaccines, i.e., DTPa/Hib and IPV.

At least 98.0% of infants had a 4-fold increase in anti-PRN antibody concentrations. At least 99.3% of infants mounted a response to PT antigen and at least 97.3% mounted a response to FHA antigen.

#### Booster vaccination study

The majority of infants remained seroprotected/seropositive against vaccine antigens prior to booster dose at the age of 18–24 months. In all groups, at least 83.9% and at least 98.9% of infants had seroprotective anti-DT, and anti-TT concentrations, respectively, at least 92.9% had seropositive concentrations of anti-PT, anti-FHA and anti-PRN antibodies, at least 89.3% were seroprotected against poliovirus 1, 2 and 3 antigens, and at least 83.1% were seroprotected against PRP (Table 2). At pre-booster vaccination, the GMC values across the 3 groups ranged from 0.154–0.175 IU/ml for anti-DT, from 0.380–0.509 IU/ml for anti-TT, from 10.3–12.2 EU/ml for anti-PT, from 12.4–14.3 EU/ml for anti-FHA, from 9.0–9.7 EU/ml for anti-PRN, from 42.6–95.7 ED_50_ for anti-poliovirus 1, 2 and 3, and from 2.275–2.674 µg/ml for anti-PRP antibodies (Table 2).

One month post-booster vaccination with DTPa/Hib and IPV vaccines, 100% of infants in the 3 groups were seroprotected against DT, TT and each poliovirus antigens, and were seropositive for anti-PT, anti-FHA, and anti-PRN antibodies. At least 99.2% of infants across the groups had seroprotective anti-PRP antibodies. The GMC values ranged from 1.227–1.504 IU/ml for anti-DT, from 4.371–4.927 IU/ml for anti-TT, from 126.8–146.2 EU/ml for anti-PT, from 120.8–124.6 EU/ml for anti-FHA, from 57.2–59.9 EU/ml for anti-PRN, from 1886.1–5438.5 ED_50_ for each anti-poliovirus, and from 27.682–49.023 µg/ml for anti-PRP antibodies (Table 2).

## Discussion

The results of the studies reported in this manuscript suggest that the 3-dose primary vaccination course of DTPa-IPV/Hib administered at 2, 3 and 4 months or 3, 4 and 5 months of age was generally well tolerated in healthy Chinese infants. In addition, the 3-dose vaccination course induced robust immune responses to all vaccine antigens that persisted up to the time of the booster vaccination with DTPa/Hib and IPV vaccines administered in the second year of life.

In the Pilot study, assessing the safety and reactogenicity of DTPa-IPV/Hib vaccine administered at 2, 3, 4 months or 3, 4, 5 months, an imbalance was observed between the study groups in terms of unsolicited symptoms which were more frequently reported in infants who received the DTPa-IPV/Hib vaccine at 2, 3, 4 months (64.0%) than in those who received the vaccine at 3, 4, 5 months (4.0%), and were mostly cases of upper respiratory tract infection and pyrexia. This increased incidence of cases in Group A was reported in early December 2009, a time when all infants had been randomized in the study but only infants in Group A had received their first dose of vaccination. One of the possible explanations for the increased reporting of unsolicited symptoms observed in Group A could be an epidemic outbreak of viral pediatric infections in the Guangxi province during the same month (December 2009). There was no increase in the reporting of unsolicited symptoms in Group B as these infants received the first dose of the vaccine after the peak of the viral epidemic had subsided. The incidence of injection site reactions and general symptoms was also higher in infants from Group A, which could potentially result from the outbreak mentioned above. In the Primary vaccination study, there were no major differences in incidence of AEs between the study groups, further supporting the hypothesis that the difference in the incidence of AEs observed in the Pilot study might have been due to the outbreak which occurred at that time. Furthermore, the reactogenicity and safety profile of the DTPa-IPV/Hib vaccine administered according to a primary 3-dose schedule was in line with previous reports.^16-19^ The incidence of AEs reported following the booster dose was consistent with the data from a recently published phase III study evaluating the safety of the booster dose of the DTPa-IPV/Hib vaccine in Vietnamese infants.^15^

The primary vaccination of Chinese infants with the combined DTPa-IPV/Hib vaccine induced robust seropositive or seroprotective antibody levels against all vaccine antigens. The immunogenicity of the DTPa-IPV/Hib vaccine in terms of antibody response was not different to that of the DTPa/Hib and IPV vaccines administered concomitantly. However, while protective levels of antibodies against diphtheria, tetanus and seropositive levels for pertussis antigens were similar in infants who received the combined DTPa-IPV/Hib and in those who received the concomitant DTPa/Hib and IPV, the protective levels of antibodies against PRP and all 3 poliovirus types appeared higher in the combined vaccine recipients. Interestingly, these results are inconsistent with findings of the previous studies, where lower anti-PRP levels were observed in infants following 3 doses of the combined vaccine as compared to infants who received separate DTPa-IPV and Hib injections.^16,19^ Nevertheless, although in these previous studies, the levels of anti-PRP antibodies were lower for the combined vaccine, all DTPa-IPV/Hib recipients achieved protective levels of antibodies. Furthermore, following a booster dose at 16–19 months of age, no differences in anti-PRP levels were observed in the combined and separate vaccine recipients.^19^ Thus, the differences between our and the previous studies might not be clinically relevant. In the Booster vaccination study reported in this manuscript, antibodies against all vaccine antigens persisted up to the time of booster vaccination, with no major differences in the percentages of seroprotected/seropositive infants and antibody GMCs. Following the booster vaccination with DTPa/Hib and IPV, all infants had seroprotective anti-DT, anti-TT, and anti-poliovirus types 1–3 levels and seropositive anti-PT, anti-FHA, anti-PRN levels, and almost all infants across the groups had seroprotective anti-PRP antibodies. Of note, the post-booster anti-PRP levels seemed higher in infants who received the combined vaccine (Group B) at 3, 4, 5 months of age compared to those who received the vaccine at months 2, 3, and 4 or who received separate administrations in the Primary study, further suggesting that decreased anti-PRP levels might not be as important in immunologically primed individuals; no further differences in antibodies against all 3 poliovirus types were observed between the groups.

A potential limitation of the studies is the open label design, which might have led to a bias in reporting of outcomes. The strengths of the studies are vaccine assessment in a representative population and a meaningful vaccination schedule.

In conclusion, the results of our studies show that the combined DTPa-IPV/Hib vaccine administered as primary 3-dose vaccination was generally well tolerated, and induced robust antibody responses to all vaccine antigens in healthy Chinese infants.

## Patients and methods

### Study design and ethics

The Pilot study was a phase III, randomized open-label, single-center study conducted at Cangwu Centre for Disease Control and Prevention (Guangxi Province, China) between December 2009 and April 2010. Healthy infants aged 60–90 d were randomized (1:1) into 2 parallel groups to receive 3 doses of DTPa-IPV/Hib vaccine either at 2, 3, 4 months of age (Group A) or 3, 4, 5 months of age (Group B).

The Primary vaccination study was a phase III, randomized, open-label study conducted at 2 centers in China, the Cangwu Centre for Disease Control and Prevention (Longxu town, Cangwu County) and Wuzhou Centre for Disease Control and Prevention (Wuzhou, Guangxi) between March 2010 and November 2010. Healthy infants aged 60–90 d were randomized (1:1:1) into 3 parallel groups to receive DTPa-IPV/Hib vaccine at 2, 3, 4 months of age (Group A), or at 3, 4, 5 months of age (Group B), or to receive DTPa/Hib and IPV vaccines at 2, 3, 4 months of age (Control group).

Infants who received 3 doses of the DTPa-IPV/Hib vaccine or DTPa/Hib and IPV administered separately (control) in the Primary study were invited to participate in a follow-up, phase IIIA, randomized, open-label Booster vaccination study, conducted between October 2011 and January 2012 to evaluate the response to booster vaccination with the same vaccines, i.e. DTPa/Hib and IPV between 18 and 24 months of age in children who received 3 doses of either DTPa-IPV/Hib or DTPa/Hiband IPV administered separately in the Primary study.

In all studies, the treatment allocation at the investigator sites was performed using a central Randomization System on Internet (SBIR, GSK Vaccines), using a minimization algorithm accounting for center.

Written informed consent was obtained from each infant's parent/legally acceptable representative prior to the performance of any study specific procedures. All three studies were conducted in accordance with Good Clinical Practice, including the Declaration of Helsinki, and all applicable local rules and regulations in China. The study protocols, the informed consent, and all documents requiring pre-approval were reviewed and approved by Institutional Review Boards. All three studies were registered with ClinicalTrials.gov (Pilot study: NCT00964028; Primary vaccination study: NCT01086423; Booster vaccination study: NCT01449812); a summary of each study protocol is available at http://www.gsk-clinicalstudyregister.com (GSK study IDs: 112065, 112584, 114386, respectively).

### Study participants

Study participants were healthy male or female infants aged 60–90 d at the time of the first study visit, who were born after a gestation period of 36 to 42 weeks, for whom written informed consent was obtained from the parent(s) or legally acceptable representative(s).

Infants were excluded if they had received any investigational drug or vaccine, or any vaccine not foreseen by the study protocol (except hepatitis B), or if they had a planned administration of such product(s) during the study period, within 30 d preceding the first dose of study vaccine, were immunosuppressed (chronic treatment with immunosuppressants, or other immune-modifying drugs since birth, or immunodeficiency), had received immunoglobulins or blood products 6 months before the study. Infants with major congenital defects, serious chronic illnesses, history of seizures or progressive neurological disease, history of allergy to any vaccine constituents, previous vaccination/history/known exposure to diphtheria, tetanus, pertussis, poliomyelitis and/or Hib disease at the time of enrolment were also excluded. Children in care were excluded for the Primary and Booster vaccination studies.

Infants were excluded from the Booster vaccination study if any of the following AEs occurred after a previous DTPa dose: encephalopathy, fever (≥40.0°C axillary temperature within 48 h of vaccination, not due to another identifiable cause), collapse or shock-like state within 48 h of vaccination, persistent crying within 48 h of vaccination and lasting ≥3 h, or seizures with or without fever occurring within 3 d of vaccination.

### Study objectives

The objective of the Pilot study was the assessment of the safety and reactogenicity of the study vaccine administered as a 3-dose primary vaccination course.

The primary objective of the Primary study was to demonstrate non-inferiority of the DTPa-IPV/Hib vaccine immunogenicity administered at 2, 3 and 4 months of age (Group A) compared to immunogenicity of the concomitant administration of DTPa/Hib and IPV vaccines at the same age (Control group), in terms of immune response to all vaccine antigens, one month after the third vaccine dose. Secondary objectives included assessment of the immune responses to the study vaccines, safety and reactogenicity.

The co-primary objectives of the Booster vaccination study included the assessment of the persistence of antibodies to all vaccine antigens at pre-booster, and immune responses to the study vaccines one month after booster vaccination. The secondary objectives included the assessment of safety and reactogenicity of the booster dose.

### Study vaccines and administration

The study vaccines were DTPa-IPV/Hib vaccine (*Infanrix-IPV/Hib*™; GSK, Belgium), DTPa/Hib vaccine, and IPV vaccine). Each 0.5 ml dose of DTPa-IPV/Hib contained ≥30 IU DT, ≥40 IU TT, 25 μg PT, 25 μg FHA, 8 μg PRN, 40 D-antigen units poliovirus type 1, 8 D-antigen units poliovirus type 2, 32 D-antigen units poliovirus type 3, 10 μg PRP conjugated to TT, 0.5 mg aluminium as salts, and ≤2.5 mg 2-phenoxyethanol. The DTPa/Hib vaccine contained the same antigen constituents as DTPa-IPV/Hib, without the poliovirus types 1–3. The IPV vaccine contained the inactivated poliovirus types 1–3 (same amount as in the DTPa-IPV/Hib), 2-phenoxyethanol, medium 199 including amino acids, formaldehyde, polysorbate 80, water for injections, and residues of neomycin sulfate.

All vaccines were administered intramuscularly into the upper side of the right (DTPa-IPV/Hib and DTPa/Hib) or left (IPV) thigh.

### Safety assessment (all studies)

Solicited local (pain, redness and swelling) and general symptoms (drowsiness, irritability, loss of appetite and fever) were recorded during a 4-day post-vaccination period. Unsolicited symptoms and SAEs were recorded up to 30 d post-vaccination. SAEs were recorded throughout the studies. SAEs related to study participation and fatal SAEs were recorded throughout the studies.

Grade 3 symptoms were defined as AEs preventing normal activity, pain upon limb movement or a spontaneously painful limb, redness and swelling >30 mm in diameter, or an axillary temperature >39.0°C, loss of appetite resulting in not eating at all, and irritability/fussiness resulting in crying that cannot be comforted or preventing normal activity. All solicited local (injection site) reactions were considered causally related to vaccination. Causality of all other AEs was to be assessed by the investigator.

Solicited and unsolicited symptoms requiring medical attention were defined as AEs resulting in hospitalization, an emergency room visit or a visit to or from medical personnel (medical doctor).

### Immunogenicity assessment (primary and booster studies)

Immunological assays in the Primary study were performed at the China National Institute for the Control of Pharmaceutical and Biological Products and in the Booster study, at the Chinese National Institute for Food and Drug Control laboratory in Beijing.

In the Primary study, immunogenicity analyses were performed in a subset of approximately 480 infants (first 160 infants in each group) before the administration of the first vaccine dose and one month after the third vaccine dose. Two blood samples were collected, one at pre-vaccination and the second, one month post-dose 3. In the Booster study, blood samples were collected from all infants at pre-booster and one month post-booster dose.

Antibodies against DT, TT, PT, PRP, FHA, and PRN were analyzed by ELISA, and antibodies against poliovirus types 1, 2 and 3 were determined by a virus micro-neutralization test adapted from the WHO Guidelines for WHO/EPI Collaborative Studies on Poliomyelitis.^20^

The ELISA assay cut-offs were: anti-PRP ≥0.15 μg/ml; anti-DT and anti-TT ≥0.1 IU/ml; anti-PT and anti-FHA ≥5 EU/ml. For anti-poliovirus types 1–3, the assay cut-off was ≥8ED_50_. As per Chinese regulatory requirements, the clinical cut-off (vaccine response) for anti-PT and anti-FHA was defined at ≥20 EU/ml. The response to PRN was defined as at least a 4-fold increase in antibody concentrations from pre to post-vaccination.

### Statistical analysis

The statistical analyses were performed using the Statistical Analysis Systems (SAS) version 9.2 and StatXact-8.1 procedure on SAS. The analysis of immunogenicity was conducted on the ATP immunogenicity cohort which included all infants who complied with study procedures and for whom immunogenicity endpoint data were available. The analyses of safety were performed on the TVC including all infants who had received at least one dose of the study vaccine.

#### Sample size

According to the Chinese Regulatory authority guidelines, the Pilot safety study required at least 20 evaluable infants. Taking into account non-evaluable participants and drop-outs, a total of 25 infants were planned to be included in each study group.

In the Primary vaccination study, 2 different sample sizes were provided, one for the subset of infants for immunogenicity evaluation, and one for the analysis of safety. The target sample size for the evaluation of immunogenicity was 480 infants (160 per group). A total of 144 evaluable infants per study group (Group A and Control Group) was required to meet all the endpoints with an overall power >90% (the overall power was computed as the sum of the individual type II errors for each endpoint, subtracted from 100%). Assuming a drop-out rate of 10%, a total of 160 infants per group were blood sampled in order to obtain the desired number of evaluable infants in the ATP cohort for analysis of immunogenicity. The target sample size for the evaluation of the safety was 990 (330 in each group) enrolled and vaccinated infants. Assuming 10% drop-out rate, the target enrolment was 990 infants in the Primary vaccination study. A total of 985 infants were expected to complete the full 3-dose primary vaccination course in the Primary vaccination study and were therefore potentially eligible for the Booster study. Assuming that approximately 80% of these infants would participate in the booster study and that 10% might be non-evaluable, the estimated sample size was 711 infants (237 per group) in the Booster study.

#### Safety analyses

Safety analyses were based on the TVC, which included only vaccinated infants and doses with documented safety data. The percentages of participants with solicited and unsolicited AEs were assessed with exact 95% CIs. SAEs and withdrawals due to AEs/SAEs were described in detail.

#### Immunogenicity analyses (Primary and Booster vaccination studies)

Immunogenicity analyses were based on the ATP cohorts for analysis of the immunogenicity, which included all evaluable infants (i.e., those meeting all eligibility criteria, complying with the procedures defined in the protocol with no elimination criteria during the study) for whom immunogenicity data were available for antibodies against at least one study vaccine antigen component at the post-primary vaccination blood sampling timepoint (Primary and Booster vaccination studies). In the Booster study, an additional cohort for analysis of antibody persistence was included, which comprised all infants who had completed their full 3-dose primary vaccination course in the Primary study, had not received an additional dose of the study vaccines since the primary study, who had no history of diseases covered by the study vaccines, and for whom serological results were available at the persistence timepoint.

Seroprotection rates and GMTs/GMCs were calculated with 95% CIs. Seropositivity was defined as an antibody concentration equal to or above the pre-specified cut-off value. The GMC calculations were performed by taking the anti-log of the mean of the log_10_ antibody concentrations transformations. Antibody concentrations below the cut-off of the assay were given an arbitrary value of half the cut-off for the purpose of GMC calculation.

#### Non-inferiority assessment (Primary study)

The non-inferiority of the immunogenicity of the DTPa-IPV/Hib vaccine compared to that of separately administered DTPa/Hib and IPV vaccines, administered in a 2, 3, 4 months schedule was demonstrated if one month after the 3-dose primary vaccination course the UL of the standardized asymptotic 95% CI for the difference between groups (Control minus Group A) in seroprotection rates against diphtheria, tetanus, Hib and 3 poliovirus types and in percentage of infants with vaccine response to PT, FHA and PRN was ≤10%.

#### Booster study

The co-primary endpoints were to assess the persistence of antibodies to all vaccine antigens before the booster dose and the immune response to the study vaccines antigens one month post-booster.

## Trademark

*Infanrix Hib, Infanrix-IPV/Hib* and *Poliorix* are trademarks of the GSK group of companies.

## Abbreviations

AE: adverse event
ATP: according-to-protocol
CI: confidence interval
DT: diphtheria toxoid
DTPa: combined diphtheria, tetanus and acellular pertussis vaccine
DTPa-IPV/Hib: combined pentavalent vaccine against diphtheria, tetanus, pertussis, poliomyelitis and Hib
ED: effective dose
ELISA: enzyme-linked immunosorbent assay
EPI: National Expanded Program on Immunization
FHA: filamentous hemagglutinin
GMC: geometric mean concentration
GMT: geometric mean titer
Hib: *Haemophilus influenzae* type b
IPV: inactivated poliovirus vaccine
IU: International units
OPV: oral poliovirus vaccine
PRN: pertactin
PRP: polyribosyl-ribitol phosphate
PT: pertussis toxoid
SAE: serious adverse event
TT: tetanus toxoid
TVC: total vaccinated cohort
UL: upper limit
WHO: World Health Organization

## References

[1] Gentile A, Bhutta Z, Bravo L, Samy AG, Garcia RD, Hoosen A, Islam T, Karimi A, Salem M, Simasathien S, et al. Pediatric disease burden and vaccination recommendations: understanding local differences. Int J Infect Dis 2010; 14:e649-58; PMID:20181506; http://dx.doi.org/10.1016/j.ijid.2009.11.006 20181506

[2] Zhang L, Wilson DP Trends in notifiable infectious diseases in China: implications for surveillance and population health policy. PLoS One 2012; 7:e31076; PMID:22359565; http://dx.doi.org/10.1371/journal.pone.0031076 22359565 PMC3281048

[3] Zhu F Pertussis booster vaccine in China. Hum Vaccin 2011; 7:272-5; PMID:21307651; http://dx.doi.org/10.4161/hv.7.2.13861 21307651

[4] Watt JP, Wolfson LJ, O'Brien KL, Henkle E, Deloria-Knoll M, McCall N, Lee E, Levine OS, Hajjeh R, Mulholland K, et al. Burden of disease caused by Haemophilus influenzae type b in children younger than 5 years: global estimates. Lancet 2009; 374:903-11; PMID:19748399; http://dx.doi.org/10.1016/S0140-6736(09)61203-4 19748399

[5] World Health Organization Polio vaccines: WHO position paper, January 2014–recommendations. Vaccine 2014; 32:4117-8; PMID:24768729; http://dx.doi.org/10.1016/j.vaccine.2014.04.023 24768729

[6] World Health Organization Certification of the Global Eradication of Poliomyelitis. Report of the fifth meeting of the Global Commission for the Certification of the Eradication of Poliomyelitis, Geneva, 9 5 2000 Available at: http://polioeradication.org/wp-content/uploads/2016/07/6report.pdf (accessed on 21 October 2016)

[7] Liang X, Zhang Y, Xu W, Wen N, Zuo S, Lee LA, Yu J An outbreak of poliomyelitis caused by type 1 vaccine-derived poliovirus in China. J Infect Dis 2006; 194:545-51; PMID:16897650; http://dx.doi.org/10.1086/506359 16897650

[8] Luo HM, Zhang Y, Wang XQ, Yu WZ, Wen N, Yan DM, Wang HQ, Wushouer F, Wang HB, Xu AQ, et al. Identification and control of a poliomyelitis outbreak in Xinjiang, China. N Engl J Med 2013; 369:1981-90; PMID:24256377; http://dx.doi.org/10.1056/NEJMoa1303368 24256377

[9] Wang HB, Fang G, Yu WZ, Du F, Fan CX, Liu QL, Hao LX, Liu Y, Zheng JS, Qin ZY, et al. An outbreak of type pi vaccine-derived poliovirus in Sichuan province, China: emergence and circulation in an under-immunized population. PLoS One 2014; 9:e113880; PMID:25503964; http://dx.doi.org/10.1371/journal.pone.0113880 25503964 PMC4263476

[10] Li R, Li CG, Li Y, Liu Y, Zhao H, Chen X, Kuriyakose S, Van Der Meeren O, Hardt K, Hezareh M, et al. Primary and booster vaccination with an inactivated poliovirus vaccine (IPV) is immunogenic and well-tolerated in infants and toddlers in China. Vaccine 2016; 34:1436-43; PMID:26873055; http://dx.doi.org/10.1016/j.vaccine.2016.02.010 26873055

[11] Li G, Zhang H, Zhou W, Ye Q, Li F, Wang H, Hou Q, Xu Y, Ma X, Tan Y, et al. Safety and immunogenicity of a diphtheria, tetanus, acellular pertussis and Haemophilus influenzae Type b combination vaccine compared with separate administration of licensed equivalent vaccines in Chinese infants and toddlers for primary and booster immunization. Vaccine 2010; 28:4215-23; PMID:20399240; http://dx.doi.org/10.1016/j.vaccine.2010.03.061 20399240

[12] Skibinski DA, Baudner BC, Singh M, O'Hagan DT Combination vaccines. J Glob Infect Dis 2011; 3:63-72; PMID:21572611; http://dx.doi.org/10.4103/0974-777X.77298 21572611 PMC3068581

[13] Public Health England Use of Infanrix®-IPV+Hib in the infant schedule: Information for Healthcare Professionals. 2015 Available at: https://www.gov.uk/government/uploads/system/uploads/attachment_data/file/417203/Infanrix-IPV-Hib_information_for_healthcare_professionals_March_2015__3__CT.pdf [Accessed on 2062016]

[14] Shao PL, Lu CY, Hsieh YC, Bock HL, Huang LM, Taiwan Infanrix-069 Study G Immunogenicity and reactogenicity of DTPa-IPV/Hib vaccine co-administered with hepatitis B vaccine for primary and booster vaccination of Taiwanese infants. J Formos Med Assoc 2011; 110:415-22; PMID:21741011; http://dx.doi.org/10.1016/S0929-6646(11)60061-2 21741011

[15] Anh DD, Van Der Meeren O, Karkada N, Assudani D, Yu TW, Han HH Safety and reactogenicity of the combined diphtheria-tetanus-acellular pertussis-inactivated poliovirus-Haemophilus influenzae type b (DTPa-IPV/Hib) vaccine in healthy Vietnamese toddlers: an open-label, phase III study. Hum Vaccin Immunother 2016; 12:655-7; PMID:26337197; http://dx.doi.org/10.1080/21645515.2015.1084451 26337197 PMC4964705

[16] Lin TY, Wang YH, Chang LY, Chiu CH, Huang YC, Tang H, Bock HL Safety and immunogenicity of a diphtheria, tetanus, and acellular pertussis-inactivated poliovirus vaccine/Haemophilus influenzae type B combination vaccine administered to Taiwanese infants at 2, 4, and 6 months of age. Chang Gung Med J 2003; 26:315-22; PMID:12934847 12934847

[17] Phua KB, Quak SH, Lim FS, Goh P, Teoh YL, Datta SK, Han HH, Bock HL Immunogenicity, reactogenicity and safety of a diphtheria-tetanus-acellular pertussis-inactivated polio and Haemophilus influenzae type b vaccine in a placebo-controlled rotavirus vaccine study. Ann Acad Med Singapore 2008; 37:546-53; PMID:18695765 18695765

[18] Dagan R, Igbaria K, Piglansky L, Melamed R, Willems P, Grossi A, Kaufhold A Safety and immunogenicity of a combined pentavalent diphtheria, tetanus, acellular pertussis, inactivated poliovirus and Haemophilus influenzae type b-tetanus conjugate vaccine in infants, compared with a whole cell pertussis pentavalent vaccine. Pediatr Infect Dis J 1997; 16:1113-21; PMID:9427455; http://dx.doi.org/10.1097/00006454-199712000-00004 9427455

[19] Halperin SA, King J, Law B, Mills E, Willems P Safety and immunogenicity of Haemophilus influenzae-tetanus toxoid conjugate vaccine given separately or in combination with a three-component acellular pertussis vaccine combined with diphtheria and tetanus toxoids and inactivated poliovirus vaccine for the first four doses. Clin Infect Dis 1999; 28:995-1001; PMID:9427455; http://dx.doi.org/10.1086/514741 10452624

[20] World Health Organization Standard Procedure for Determining Immunity to Poliovirus using the Microneutralization Test (WHO/EPI/GEN 93.9) 1993. Available at: http://apps.who.int/iris/bitstream/10665/70486/1/WHO_EPI_GEN_93.9_eng.pdf [Accessed on 1542016]

